# Sustainable Extraction of *Actinostemma lobatum* Kernel Oil by 2-Methyltetrahydrofuran: A Comparative Study on Physicochemical Properties and Bioactive Compounds Against Petro-Sourced Solvents

**DOI:** 10.3390/foods14101682

**Published:** 2025-05-09

**Authors:** Liyou Zheng, Hongyan Guo, Haozhi Song, Miao Yu, Mengxi Xie, Sameh A. Korma, Tao Zhang

**Affiliations:** 1 School of Biological and Food Engineering, Anhui Polytechnic University, Wuhu 241000, China; zhengliyou@ahpu.edu.cn (L.Z.); 2106032@mail.ahpu.edu.cn (H.G.);; 2 Institute of Food and Processing, Liaoning Academy of Agricultural Sciences, Shenyang 110161, China; jannytiti@163.com (M.Y.); moor1112@163.com (M.X.); 3 Department of Food Science, Faculty of Agriculture, Zagazig University, Zagazig 44519, Egypt; 4 School of Food Science and Engineering, South China University of Technology, Guangzhou 510641, China; 5 College of Food Science and Technology, Huazhong Agricultural University, Wuhan 430070, China

**Keywords:** *Actinostemma lobatum* kernel oil, 2-Methyloxolane, harvest year, solvent type, bioactive compounds, thermal property, tocotrienols

## Abstract

This study aims to evaluate the effect of extraction solvent type on the physicochemical properties and bioactive compounds of *Actinostemma lobatum* Maxim. kernel oil for two successive harvest years. Oils were extracted using the bio-based solvent 2-methyltetrahydrofuran (2-MeTHF) and conventional petroleum-derived solvents (*n*-hexane and 2-methylpentane). Results indicated that 2-MeTHF achieved significantly higher oil yields (27.60% in 2021 and 29.77% in 2022) compared to *n*-hexane and 2-methylpentane. Unfortunately, 2-MeTHF-extracted oils exhibited greater susceptibility to oxidation, displaying elevated levels of primary and secondary oxidation products relative to other solvents. Meanwhile, 2-methylpentane-extracted oil showed a relatively high oxidative stability index. In addition, differential scanning calorimetry results also aligned with the oxidative status. Further variance analysis revealed that the harvest year exerted a more pronounced impact on fatty acid and triacylglycerol profiles than the solvent type. Additionally, tocopherols and tocotrienols were abundant, with β- and δ-tocopherols predominating. 2-MeTHF-extracted oils harvested in 2022 contained the highest total tocols (1118.83 mg/kg) among all samples. Also, phytosterols were detected, with β-sitosterol constituting the predominant compound. Furthermore, the 2-MeTHF-extracted oils contained higher *β*-carotene contents compared to other samples. These above findings concluded that 2-MeTHF is a good alternative to conventional solvents for extracting of *A. lobatum* kernel oil.

## 1. Introduction

*Actinostemma lobatum* Maxim. is widely distributed across Asia, particularly in China and Korea [1]. Its kernel contains approximately 47% oil by weight, dominated by linoleic acid (43–44%) and oleic acid (37–38%). With the increasing global demand for vegetable oils in the last few decades [2], *A. lobatum* kernel oil appears to be a viable and sustainable alternative. According to our previous study, this kernel oil also contains significant concentrations of fat-soluble bioactive compounds such as squalene, tocopherols, and phytosterols [1], positioning *A. lobatum* kernel as a promising oilseed for edible oil production [3,4]. It is noteworthy that *A. lobatum* kernel oil contains a relatively high content (2327–2975 mg/kg) of squalene, which is within the content range (1100 to 8390 mg/kg) in olive oil [1,5].

Though mechanical pressing and solvent extraction are common methods for oil production [6,7], solvent extraction achieves superior yields (up to 98% recovery) and is therefore industrially favored [8]. *n*-Hexane remains the dominant solvent due to its chemical stability, high oil selectivity (95–97% efficiency), low energy requirements, and favorable boiling point (69 °C) [8,9]. Additionally, a *n*-hexane-defatted meal is odorless and has a low residual oil content, making it highly marketable [10]. However, *n*-hexane has recently aroused growing concerns because of negative environmental impacts and toxicity [11]. On the one hand, it is classified as a hazardous air pollutant by the United States Environmental Protection Agency, leading to strict regulations in the United States and the European Union [8,12,13,14]. On the other hand, it is registered as a category 2 reprotoxic, carcinogenic, mutagenic substance, as well as a category 2 aquatic chronic toxic substance under the REACH Regulation [7,15]. Consequently, the search for environmentally friendly alternatives to *n*-hexane in oil extraction is gaining increasing attention [9].

Industrial oil extraction processes have been optimized for *n*-hexane since the 1950s. Therefore, only solvents with similar physicochemical properties are suitable alternatives in the oil industries, which rely on large-scale processing facilities [16]. 2-Methylpentane (2-MP) and 2-methyltetrahydrofuran (2-MeTHF) are probably two viable alternatives (Table S1). 2-MP is a branched-chain alkane and a structural isomer of *n*-hexane. It was reported to have lower neurotoxicity than *n*-hexane, and no literature reports were found indicating that chronic exposure to 2-MP has caused toxicity, cancer, or birth defects in humans or that it affects reproduction [17]. Therefore, 2-MP tends to be much safer than industrial *n*-hexane [18]. 2-MeTHF, also known as 2-methyloxolane, is a bio-based solvent and a potential substitute for petroleum-derived solvents [7]. In particular, 2-MeTHF is a green solvent characterized by low toxicity, biodegradability, and minimal environmental impact [19]. It emerges as a promising alternative to conventional petroleum-based solvents such as *n*-hexane [7,19,20,21]. 2-MeTHF has been successfully applied for the extraction of *Litsea cubeba* kernel oil [9], rapeseed oil [15], yeast *Yarrowia lipolytica* lipid [22], and black soldier fly oil [23]. No information was found on *A. lobatum* kernel involved 2-MeTHF.

To our knowledge, this work provides the first comparative analysis of 2-MeTHF and 2-MP for *A. lobatum* kernel oil extraction for two successive harvest years, addressing a critical gap in sustainable solvent research. The extracted oils were then systematically compared with those obtained using the conventional solvent (*n*-hexane) in terms of physicochemical properties (oil yield, oil color, fatty acid and triacylglycerol profiles, oxidative status, oxidative stability, and thermal properties) and bioactive compounds (tocopherols, tocotrienols, phytosterols, and *β*-carotene).

## 2. Materials and Methods

### 2.1. Materials

Fruits of *A. lobatum* were collected from Shenshan National Wetland Park (Wuhu, China) in October 2021 and 2022. The moisture content of the kernels was determined according to the AOCS Official Method Ba 2a-38, with values of 6.95 ± 0.03% (2021) and 7.23 ± 0.06% (2022). Both values met the requirements of AOCS Official Method Am 2-93, which specifies that kernel moisture content must be less than 10%. After manual removal of impurities, the kernels were stored at −20 °C until further analysis.

Standards for 37 fatty acid methyl esters, four tocopherols (*α*-, *β*-, *γ*-, and *δ*-tocopherol, purity > 98%), four tocotrienols (*α*-, *β*-, *γ*- and *δ*-tocotrienols, purity > 98%), 2-olein monoacylglycerol, 1,2-diolein and 1,3-diolein diacylglycerols, oleic acid, and cholestan-3-ol were purchased from Sigma-Aldrich Chemical Co., Ltd. (Shanghai, China). 2-MP (purity > 98%) and 2-MeTHF (purity > 99%) were obtained from Shanghai Aladdin Biochemical Technology Co., Ltd. (Shanghai, China). Other reagents and solvents were supplied by Sinopharm Chemical Regent Co., Ltd. (Shanghai, China).

### 2.2. Oil Content

Oil content of the *A. lobatum* kernel was determined using a Soxhlet extractor (SOX606, Hanon Advanced Technology Group Co., Ltd., Qingdao, China). Petroleum ether (boiling range: 30–60 °C) was used as the solvent, with an extraction temperature of 70 °C and a duration of 10 h. Oil content (%) was calculated as the ratio of extracted oil mass to the initial sample mass. The oil contents of *A. lobatum* kernel harvested in 2021 and 2022 were 42.08 ± 1.22% and 39.97 ± 2.15%, respectively.

### 2.3. Oil Extraction

Approximately 150 g of *A. lobatum* kernels were ground using a laboratory mill (800C, Yongkang Red Sun Mechanical and Electrical Co., Ltd., Jinhua, China) to a maximum particle size of 0.85 mm. The extraction solvents including *n*-hexane, 2-MeTHF, and 2-MP, and corresponding oil samples obtained from 2021 and 2022 were referred to as Hexane-2021, 2-MeTHF-2021, 2-MP-2021, Hexane-2022, 2-MeTHF-2022, and 2-MP-2022. Table S1 lists the properties of these solvents.

The extraction process was conducted by mixing 25 g of ground samples with 250 mL of extraction solvents. The mixtures were placed into Erlenmeyer flasks and extracted in a horizontal shaking bath (SHA-B, Shanghai Lichen Instrument Technology Co., Ltd., Shanghai, China) at 60 °C for 4 h. The solvents were then removed using a rotary evaporator (R-1001VN, Zhengzhou Greatwall Scientific Industrial and Trade Co., Ltd., Zhengzhou, China) at 50 °C under reduced pressure. The obtained oil was cooled in a desiccator, and then stored at −20 °C in dark glass bottles for further analyses.

### 2.4. Physicochemical Properties Analyses

#### 2.4.1. Oil Yield

Oil yield was calculated as the ratio of the mass of extracted oil to the mass of the test sample. The test was performed in duplicate.

#### 2.4.2. Oil Color

The color values R and Y (R, red; Y, yellow) of the oil samples were determined according to AOCS Method Cc 13b-45. The oil samples were placed into a 25.4 mm optical path spectrophotometer cell and then tested according to the AOCS RY method. The color value was expressed as 5R + Y, according to the previous method [24].

#### 2.4.3. Study of the Oxidative Status of Oil

The following parameters in crude oils were evaluated according to standard AOCS methods: acid value (AV), Cd 3d-63; *p*-anisidine value (*p*-AnV), Cd 18-90; K_232_ and K_268_, and Ch 5-91.

#### 2.4.4. Oxidative Stability Index (OSI)

The OSI was analyzed based on the procedures described by [25]. Briefly, the accelerated oxidation was performed using a Rancimat instrument (Model 892, Metrohm, Herisau, Switzerland) with some modifications. The oil samples (3 g) were heated at 110 °C (ΔT = 1.6) and, meanwhile, 20 L h^−1^ of the cleaned and dried air was bubbled into the hot sample. The OSI, expressed in hours, was obtained to describe the oil sample stability.

#### 2.4.5. Fatty Acid Composition

Oil samples at a concentration of 25 mg mL^−1^ (50 mg oil dissolved in 2 mL *n*-hexane) were first prepared and then methyl-esterified by adding 0.5 mL of 2 mol L^−1^ potassium hydroxide in methanol, followed by vigorous shaking for 5 min. Then, 1 mL of a saturated NaCl solution was added, followed by collecting the upper layer for later gas chromatography analysis. The analysis procedures followed by the method [26]. Then, the nutritional quality of the extracted oils was assessed based on the following indices [27,28]:

The atherogenicity index (AI):

$$\mathrm{AI}=\left(C12:0+4\times C14:0+C16:0\right)/\left(\sum \mathrm{UFA}\right)$$

The thrombogenicity index (TI):

$$\mathrm{TI}=\left(C14:0+C16:0+C18:0\right)/\left(0.5\times \sum \mathrm{UFA}+2.5\times \sum \text{n-3PUFA}+\text{n-3PUFA}/\text{n-6PUFA}\right)$$

The hypo- and hyper-cholesterolemic index (HH):

$$\mathrm{HH}=\left(C18:1+\sum \text{n-6PUFA}\right)/\left(C12:0+C14:0+C16:0\right)$$

#### 2.4.6. Triacylglycerol Composition

Triacylglycerol composition was analyzed by an ultra-performance convergence chromatography (UPC^2^) system coupled with quadrupole time-of-flight mass spectrometry (Q-TOF-MS), as reported by [29]. The contents of different triacylglycerol molecules were reported as the relative proportions.

#### 2.4.7. Thermal Analysis by Differential Scanning Calorimetry (DSC)

**Figure 1:**
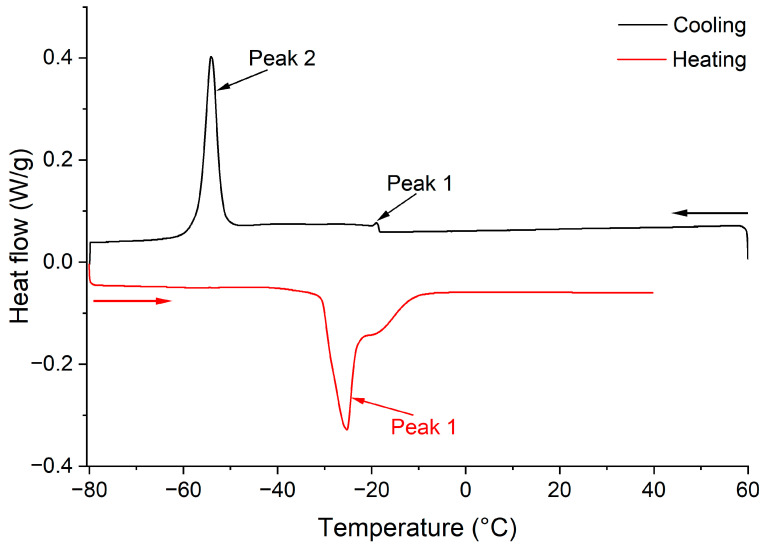
DSC curves of melting and crystallization of oil sample.

Thermal analysis was performed using a DSC 25 differential scanning calorimeter (TA Instruments Japan Inc., Tokyo, Japan) equipped with an RCS90 refrigerated cooling system, following the reported method [30] with slight modifications. The DSC instrument was enthalpy-calibrated with indium (28.22 J/g) at 156.58 °C. *A. lobatum* kernel oil (around 5.0 mg) was placed in a standard DSC aluminum pan with hermetic sealing, while an empty sealed DSC aluminum pan was used as the reference. The oil samples were subjected to the following temperature program: the sample was held at 60 °C isotherm for 10 min to eliminate the thermal history of the samples, then cooled at 2 °C/min to −80 °C and held for 2 min. The sample was then heated from −80 to 40 °C at the same rate. During the tests, nitrogen (99.99% purity) was used as the purge gas at a flow rate of 50 mL/min. The DSC parameters of melting and crystallization curves were determined to characterize each sample. The onset temperature (*T_on_*, °C), the offset temperature (*T_off_*, °C) (points at which the extrapolated leading edge of the endotherm/exotherm intersects with the baseline), peak temperature, peak height (h), and the enthalpy (ΔH, J/g) of thermal transition (melting and crystallization) of *A. lobatum* kernel oils were determined from the acquired thermograms (Figure 1). All DSC measurements were conducted in duplicate.

#### 2.4.8. Fourier Transform Infrared Spectroscopy (FT-IR)

An FT-IR spectrometer (IRPrestige-21, Shimadzu Tokyo Innovation Plaza, Kawasaki, Japan) was used to obtain the IR spectrum of *A. lobatum* kernel oil. The data were collected over a spectral range of 4000–400 cm^−1^ (440 scans/sample or background) at 4 cm^−1^ resolution in reflectance mode [27].

### 2.5. Bioactive Compounds Analyses

#### 2.5.1. Tocopherol and Tocotrienol

The tocopherol and tocotrienol isomers were examined using a high-performance liquid chromatography system (LC-20AT, SHIMADZU (China) Co., Ltd., Shanghai, China) equipped with a fluorescence detector (SHIMAZU RF-20Axs). The excitation wavelength was set at 294 nm, and the emission wavelength was set at 328 nm, following the standard GB/T 26635—2011/ISO 9936:2006 [31] with minor modifications. In short, oil samples weighing 50 mg (to an accuracy of 0.0001 g) were dissolved in a 5 mL brown volumetric flask, and 20 µL of the sample was injected into the system. The separation was performed on a LiChrospher^®^ 100 DIOL HPLC column (5 µm, 4.6 mm × 250 mm, Merck KGaA, Darmstadt, Germany) coupled with a MANU-CART^™^ NT HPLC cartridge holder, using a mixture of *n*-heptane and tetrahydrofuran (3.85/96.15, *v*/*v*) as the mobile phase at a flow rate of 1.0 mL min^–1^.

#### 2.5.2. Phytosterols

Phytosterols were determined using a gas chromatography-mass spectrometry system (Thermo Fisher, Waltham, MA, USA), as described by [26]. The content was quantified using the internal standard method.

#### 2.5.3. *β*-Carotene

*β*-carotene content was determined following BS 684-2.20 using the Lovibond Model Fx Tintometer (Tintometer Group, Amesbury, UK). The oil samples were placed into a 25.4 mm optical path spectrophotometer cell and then tested according to the AOCS RY and β-carotene method.

### 2.6. Statistical Analyses

All experiments were conducted in triplicate or duplicate, and the results are presented as average values ± standard deviation (SD). Statistical analyses were performed using SPSS version 19.0 for Windows (SPSS Inc., Chicago, IL, USA). Comparisons between two means were conducted using unpaired Student’s *t*-tests, while comparisons among three or more means were performed using ANOVA followed by multiple comparisons with Duncan’s multiple range test. Differences were considered significant at *p* < 0.05. Hierarchical cluster analysis, linear correlation analysis, and the figures were mainly constructed by OriginPro 2021 (OriginLab Corporation, Northampton, MA, USA).

## 3. Results and Discussions

### 3.1. Physicochemical Properties

#### 3.1.1. Oil Yield

A higher oil yield is essential to meet the growing demand for vegetable oils. After quantitatively extracting *A. lobatum* kernels for 4 h using *n*-hexane, 2-MeTHF, and 2-MP, oil yields were determined gravimetrically. The effects of different solvents and harvest years on the extraction yield of *A. lobatum* kernel oils are illustrated in Table 1. Generally, oil yield ranged from 22.74% to 29.77%, and significant differences were detected among solvent types and harvest years (*p* < 0.05). The oil recoveries were roughly equivalent with our previous result using Soxhlet extraction with petroleum ether [1]. Since *A. lobatum* kernel contains approximately 40% oil, the combination of organic solvent extraction and mechanical pressing extraction processes are recommended at semi-industrial and industrial scales, i.e., continuous mechanical pressing with continuous organic solvent extraction, and batch hydraulic pressing with solvent extraction [32]. Meanwhile, further works are warranted to explore the application of novel extraction technologies such as aqueous extraction, supercritical fluid extraction, and subcritical fluid extraction for *A. lobatum* kernel oil. Analysis of variance revealed that both solvent type and harvest year significantly affected the oil yields (*p* < 0.05) in the present study.

From the perspective of solvent type, 2-MeTHF remarkedly extracted the most lipids from *A. lobatum* kernels (27.60% for 2021 and 29.77% for 2022), ranking 2-MeTHF > *n*-hexane > 2-MP in both years. The results were in accordance with the results reported in [7,8,12,33]. We speculate that the differences in oil yield may be attributed to the characteristics of *n*-hexane, 2-MeTHF, and 2-MP. For example, the partition coefficient of 2-MeTHF is 1.85, whereas that of *n*-hexane and 2-MP is 4.0 and 3.20, respectively, indicating that 2-MeTHF is a more polar solvent [12]. Consequently, 2-MeTHF is more likely to extract a higher fraction of more polar lipids, such as phospholipids, diglycerides, and free fatty acids [16]. Unfortunately, 2-MP does not seem to be an ideal alternative for *n*-hexane in terms of oil yield. From the aspect of harvest year, the oil yield of *A. lobatum* kernels harvested in 2022 was higher than that of 2021 whatever the solvents, namely Hexane-2022 > Hexane-2021, 2-MeTHF-2022 > 2-MeTHF-2021, and 2-MP-2022 > 2-MP-2021. In addition, even though the oil yield in 2022 decreased by 2-MP compared with *n*-hexane, it remained higher than that of *n*-hexane in 2021.

#### 3.1.2. Oil Color

The crude oils extracted with *n*-hexane and 2-MP exhibited a light-yellow color, while oil extracted with 2-MeTHF had a reddish color. The color results (5R + Y) of *A. lobatum* kernel oil are shown in Table 1. Significant differences in oil color were observed among solvents (*p* < 0.05), with 2-MeTHF-extracted oil displaying the highest color values (129.25 and 120.00 for 2021 and 2022, respectively). The oil color was ranked as 2-MeTHF > 2-MP > *n*-hexane for both harvest years. High color value of 2-MeTHF extracted oil was also reported [7,16]. The results probably stem from 2-MeTHF’s high recovery of individual and total carotenoids in different conformations, polarities and properties [20]. The *β*-carotene contents of the oil samples also align with these color results. It was reported that 2-MeTHF enables the recovery of both carotenes and xanthophylls, like astaxanthin or lutein [16], which can account for higher red and yellow color values. Further analysis of variance similarly revealed that both solvent type and harvest year had a significant impact on the oil color (*p* < 0.05), and the solvent type showed a lower *p* value compared to the harvest year.

#### 3.1.3. Oxidative Status

##### K232 and K268

The effect of solvent type and harvest year on the K_232_ and K_268_ values of *A. lobatum* kernel oils is illustrated in Table 1. In general, significant differences were observed in the values of K_232_ and K_268_ among the tested samples (*p* < 0.05). For instance, the levels of K_232_ and K_268_ in 2-MeTHF-2021 were 11.58 and 2.65, while 2-MeTHF-2022 with 10.14 and 2.46, respectively. These results were considerably higher than those in other oil samples (*p* < 0.05), ranking as 2-MeTHF > 2-MP > Hexane for both harvest years. Similar results have been reported in soybean oil and sesame oil extracted with 2-MeTHF and *n*-hexane [7,8]. The high levels of K_232_ and K_268_ in 2-MeTHF-extracted oils indicated that oils were in a more advanced deterioration stage compared to those extracted with *n*-hexane and 2-MP, though this could also be attributed to the residual 2-MeTHF or its peroxides [7,34]. In addition, the harvest year did significantly affect the levels of K_232_ and K_268_ in oils (*p* < 0.05). Oils harvested in 2021 were prone to be with higher values when compared to K_232_ and K_268_ of oils harvested in 2022 within the same solvent. Further variance analysis revealed that both the solvent type and harvest year showed significant effects on the K_232_ and K_268_ (*p* < 0.05), with the solvent having lower *p* values than the harvest year.

##### AV and *p*-AnV

As shown in Table 1, the solvent type significantly influenced the AV and *p*-AnV values of *A. lobatum* kernel oils (*p* < 0.05). The highest AV appeared in the *A. lobatum* kernel oil extracted with 2-MeTHF, reaching 3.75 and 2.57 mg KOH/g for the 2021 and 2022 harvest years, respectively. As previously mentioned, 2-MeTHF is a more polar solvent compared to 2-MP and *n*-hexane. Increased solvent polarity may elevate AVs by disrupting oil bodies and extracting more free fatty acids [8,16]. A similar trend was also reported [7]. However, no obvious trends were found in AVs among oils extracted with *n*-hexane and 2-MP. Both the solvent type and harvest year remarkably affected AV (*p* < 0.05).

Oil extracted with 2-MeTHF showed significantly higher *p*-AnV compared to other oil samples for both harvest years (*p* < 0.05). Furthermore, oils harvested in 2022 showed significantly higher *p*-AnV than those harvested in 2021 (*p* < 0.05). More polar substances like free fatty acids and trace amounts of water in 2-MeTHF extracted oils may trigger lipid hydrolysis and oxidation. However, opposite results were also reported [7]. The environmental conditions and solvent type may affect the results. Overall, these results suggest that 2-MeTHF-extracted oils are initially in a more deteriorated state than conventionally extracted oils [7], and require further refining after extraction to maintain the oil quality.

#### 3.1.4. OSI

The OSI (h) value indicates the oxidative stability of the samples, with higher value suggesting greater resistance to oxidation. In 2021, the OSI values varied significantly depending on the solvent type, with 2-MP showing the highest oxidative stability (41.50 h), followed by 2-MeTHF (17.13 h) and *n*-hexane (11.12 h). In 2022, the OSI values showed a different trend compared to 2021 as shown in Table 1. Great variations were observed among OSI values. The oils extracted with 2-MeTHF had high levels of primary (K_232_ and K_268_) and secondary (*p*-AnV) oxidation products, making them prone to oxidation with a low OSI value. However, comparable or high OSI values were reported for 2-MeTHF-extracted oils compared to that of oils extracted by *n*-hexane [7,8,35]. The purity of 2-MeTHF may not be exactly true, and needs further investigation. The significant decline in the OSI value for 2-MeTHF-2022 is particularly noteworthy and warrants further investigation into the contents of polyphenols and phospholipids to try to explain these great variations. Additionally, the consistently high performance of 2-MP suggests that it seems to be a more reliable solvent to preserve oxidative stability during the extraction process.

Considering the above oxidative status (including K_232_, K_268_, AV, and *p*-AnV), future studies should focus on optimizing extraction conditions and ensuring high-quality solvents to achieve consistent and high oxidative stability in the extracted products. The oxidative status of oils extracted with 2-MeTHF suggests that refining or addition of antioxidants may be necessary.

#### 3.1.5. Fatty Acid

Fatty acid profile of *A. lobatum* kernel oil is presented in Table 1 and Figure S1. The kernel oil is mainly composed of palmitic (C16:0), oleic (C18:1), linoleic (C18:2), myristic (C14:0), and stearic (C18:0) acids, which together make up more than 95% of the total fatty acids. Other small amounts (less than 1%) of fatty acids include lauric (C12:0), palmitoleic (C16:1n9), margaric (C17:0), arachidic (C20:0), eicosenoic (C20:1), linolenic (C18:3n3), behenic (C22:0), and lignoceric (C24:0) acids. These results were consistent with former results reported by [1].

No significant differences were observed among the oil samples extracted with different solvents (*p* > 0.05). Figure S1 shows that oils extracted with *n*-hexane and 2-MP were clustered into one group due to their similar fatty acid profile for both harvest years. Furthermore, the fatty acid profile varied significantly depending on the harvest year (*p* < 0.05). In oils obtained from kernels harvested in 2021, oleic acid was the most abundant fatty acid, followed by linoleic acid. However, in the 2022 samples, the proportions of oleic and linoleic acids were reversed, which aligned with previous findings [1]. This variation may be attributed to differences in environmental conditions (such as drought and temperature) and the maturation stage.

The high polyunsaturated fatty acid in *A. lobatum* kernel oil suggests its potential as a health-promoting agent. Some evidence indicates that AI can be used as a risk factor or predictor for cardiovascular diseases, with higher AI values signifying greater risk [36]. The AI value of *A. lobatum* kernel oil was 0.29 close to that of corn oil (AI = 0.27), wheat germ oil (AI = 0.28), and grape seed oil (AI = 0.28), but significantly higher than almond oil (AI = 0.07), olive oil (AI = 0.16), and soybean oil (AI = 0.18) [36,37]. The TI characterizes the thrombogenic potential of fatty acids, indicating the tendency to form clots in blood vessels [38]. The TI range of *A. lobatum* kernel oil (0.26–0.27) was similar to that of grape seed oil (TI = 0.28) but significantly lower than poppy seed oil (TI = 0.32) and walnut oil (TI = 0.35) [37]. Nutritionists emphasize that high HH value is beneficial for human health [38]. The HH value of *A. lobatum* kernel oil (6.98–7.20) was much lower than that of camelina oil (HH = 11.2−15.0) and raspberry seed oil (HH = 18.8−52.8) but significantly higher than most reported oils [38]. In summary, the low AI and TI values and the high HH value of *A. lobatum* kernel oil indicate its high nutritional quality.

#### 3.1.6. Triacylglycerol Analysis

**Figure 2:**
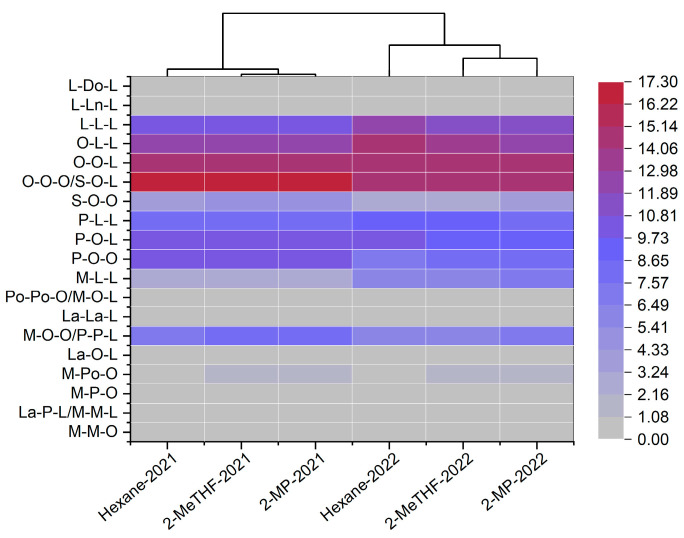
Heatmap with dendrogram of the oils using the triacylglycerol data (La, C12:0; M, C14:0; P, C16:0; Po, C16:1; S, C18:0; O, C18:1; L, C18:2; Ln, C18:3; Do, C22:0).

As expected from the fatty acid composition, most of all the triacylglycerols included oleic (C18:1) and linoleic (C18:2) acids in their structure (Figure 2). Nineteen TAG species were found in the kernel oils, similar to our previous results [1]. Four major triacylglycerols (C18:1-C18:1-C18:1/C18:0-C18:1-C18:2, C18:1-C18:1-C18:2, C18:1-C18:2-C18:2, and C18:2-C18:2-C18:2) account for 52.2−56.5% of the total triacylglycerols. The determination of the similarity in the triacylglycerol compositions as shown in Figure 2, and hierarchical cluster analysis illustrated a clear cluster distribution of oils containing the same triacylglycerols. Two clusters were obtained depending mainly on the harvest years. The main reason was the different fatty acid profiles (especially the oleic and linoleic acids) as shown in Table 1. As for the solvents, oils extracted by *n*-hexane and 2-MP showed similarity in the triacylglycerol profiles as they were clustered into one group.

#### 3.1.7. DSC Melting and Crystallization Profiles

**Figure 3:**
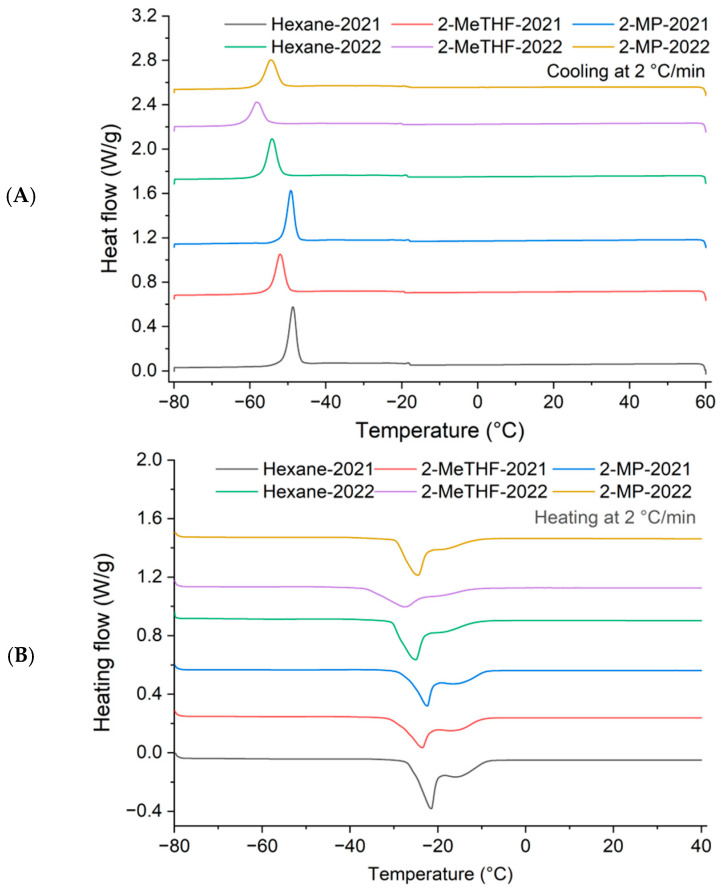
Crystallization curve (**A**) and melting curve (**B**) of *A. lobatum* kernel oil.

The thermal behavior of vegetable oils is usually described by their melting and crystallization characteristics [36]. These thermal properties are profoundly affected by the composition of triacylglycerol profiles, which are determined by the fatty acid composition and their distribution within triacylglycerols [30]. In this study, the thermal properties of *A. lobatum* kernel oil were initially determined based on the melting and crystallization profiles (Figure 3). The peaks detected during the cooling and heating processes were associated with different triacylglycerol groups in *A. lobatum* kernel oil, including high unsaturated triacylglycerol (UUU), moderate unsaturated triacylglycerol (SUU), and low unsaturated triacylglycerol (SSU). The major peaks (at low temperatures) in the cooling and heating profiles corresponded to the predominant triacylglycerols (UUU, ~54%), especially LLL (~10%). The smaller peaks (at high temperatures) were attributed to SUU and SSU [30,36].

In the cooling curves (Figure 3A), two exothermal peaks (1 and 2) were detected. The peak temperature of the major peak (2) for all samples ranged from −48.63 °C to −57.89 °C, with T_on_ and enthalpy values varying between −44.11 °C and −55.03 °C and from 26.71 J/g to 41.57 J/g, respectively. The small exothermic peak (1) occurred at a high temperature, with *T_on_* values between −19.64 °C and −17.89 °C. Generally, the crystallization of vegetable oils is a gradual process, beginning with the formation of microcrystalline structures that subsequently transform into macrocrystalline forms, leading to a solid-like consistency [6]. In this study, the *T_on_* of the small peak (peak 1) precisely indicated the temperature at which microcrystals start forming through the stacking of the ‘‘bent tuning fork’’ conformation during the initial process. As cooling progressed, the solvation power of *A. lobatum* kernel oil matrix decreased, causing the oil to transition from a highly viscous fluid to a solid phase, as marked by a *T_on_* of the primary freezing peak (peak 2). The DSC parameters for the crystallization curves of *A. lobatum* kernel oil are illustrated in Table 2.

Table 3 shows the DSC parameters of the melting curves for *A. lobatum* kernel oil (Figure 3B). A prominent endothermic peak (1) was detected, with a sharp melting peak temperature ranging from −27.58 °C to −21.76 °C and enthalpy values between 9.29 J/g and 20.94 J/g. A shoulder on the melting curve was observed at about −15 °C. Such shoulders are commonly observed in DSC melting curves of vegetable oils due to the presence of diverse triacylglycerols and various triacylglycerol polymorphic forms (*α* and *β*), resulting in melting over a wide temperature range [39,40].

As for the extraction solvents, solvent type showed a significant effect on the DSC parameters (*p* < 0.05). For instance, 2-MeTHF-extracted oils had a significantly lower peak temperature, *T_on_*, and *T_off_* compared to the other solvents (*p* < 0.05). Specifically, the increased secondary oxidation products (*p*-AnV), AV (Table 1), and partial glycerides likely contributed to the formation of polymorphic crystals that were structurally distinct and less stable than those in pure oil [41]. These factors may also have hindered the transition or rearrangement of triacylglycerol polymorphic crystals [41,42]. Similarly, significantly lower peak height and enthalpy were also observed in both the crystallization curve and melting curve for 2-MeTHF-extracted oils in comparison with oils extracted by *n*-hexane and 2-MP. The reduction in the enthalpy and peak height of 2-MeTHF-extracted oils may be attributed to the disappearance of triglycerides and the formation of degradation products that do not crystallize in the scanning temperature range [43]. In addition, significant differences (*p* < 0.05) in peak temperature, *T_on_*, and *T_off_* of the cooling and heating curves were shown between the oils extracted in 2021 and 2022. The high unsaturation degree of fatty acid (Table 1) and triacylglycerol profiles (Figure 2) in 2022 can account for the results [36].

#### 3.1.8. FT-IR Analysis

The FT-IR fingerprint spectra of *A. lobatum* kernel oils, displaying twelve peaks in the mid-infrared region of 4000–400 cm^−1^, are shown in Figure S2. The positions of the peak maxima reflect the functional groups present in *A. lobatum* kernel oils [27]. In this study, the spectra of the oil samples showed many similarities among absorbance peaks. The explanations of these peaks were provided in Section S1 of Supplementary Materials. Overall, no obvious trends in FT-IR spectra were observed for different solvent types and harvest years.

### 3.2. Bioactive Compounds Analyses

#### 3.2.1. Tocopherol and Tocotrienol

Tocopherol and tocotrienol are the most abundant natural antioxidants and serve as the primary antioxidants in vegetable oils [1]. For the first time, tocotrienol analysis of *A. lobatum* kernel oils was conducted, detecting *α*-, *γ*-, and *δ*-tocotrienols. Table 4 shows that *α*-tocotrienol is the most abundant tocotrienol form in *A. lobatum* kernel oils, with its content in the range from 5.55 to 38.76 mg/kg. In addition, four homologs of tocopherols were detected in *A. lobatum* kernel oil. As presented in Table 4, *β*- and *δ*-tocopherols were the major tocopherol forms, followed by *α*- and *γ*-tocopherols, consistent with our previous results [1]. In detail, *β*- and *δ*-tocopherols accounted for about 75.5−82.3% of the total tocols. α-Tocopherol content (126.07−178.01 mg/kg) and *γ*-tocopherol content (34.93 to 44.72 mg/kg) accounted for 12.0−17.7% and 4% of the total tocols, respectively. The total tocols (888.12−1152.85 mg/kg) were roughly equivalent to those in corn germ oil and soybean oil [6], but significantly higher than the values we previously reported [1].

Regarding the solvent type, 2-MeTHF extracted comparable levels of total tocols to *n*-hexane, with no significant differences (*p* < 0.05). The current findings were consistent with the findings reported in previous studies [7,15]. However, Trad et al. [8] reported that sesame oils obtained using 2-MeTHF exhibited higher tocopherol content in comparison to *n*-hexane. When it comes to the harvest year, oils from 2022 oilseeds showed significantly higher contents of total tocols than those from 2021 (*p* < 0.05). Similarly, harvest year was also reported to show a significant effect on the contents of tocopherol in almond kernels [44]. These results suggest the role of climatic and stress conditions as well as the soil quality on the distribution of tocopherol homologues [44,45]. The analysis of variance, considering three extraction solvents and two harvest years, showed that *γ*-tocopherol was significantly affected by the solvent type, while the harvest year effect was significant for *α*-, *β*-, *δ*-tocopherols and total tocols (*p* < 0.05).

#### 3.2.2. Phytosterols

Phytosterols are broadly applied in functional foods, supplements, and pharmaceutical products due to their ability to lower LDL-cholesterol [46]. The phytosterol composition in *A. lobatum* kernel oils is shown in Table 4. Ten kinds of phytosterols were detected, with *β*-sitosterol being the major phytosterol (59.6–65.9% of total sterols, 1210.0−1356.7 mg/kg). In addition, campesterol, stigmastenol, stigmastadienol, and Δ^5^-avenasterol were also present at significant levels, roughly accounting for 27.5−33.8% of total sterols.

Solvent type had a significant effect on the phytosterol contents. For instance, oils extracted using 2-MeTHF possessed lower contents of phytosterols compared to those extracted with 2-MP and *n*-hexane. These results aligned with Claux et al. [7]. However, Trad et al. [8] reported that 2-MeTHF extracted higher sterols content than *n*-hexane. No significant differences were found between 2-MP and Hexane groups (*p* > 0.05). The polarity and chemical structures of the main sterols present in the oils may explain these variations. Regarding cultivation year, oilseeds from 2021 exhibited higher contents of phytosterols than those from 2022. The results of the variance analysis showed that the sterol levels were significantly impacted by the harvest year, with the exception of brassicasterol and campesterol, and by the solvent type, affecting most sterol contents except campesterol, campestanol, and Δ^7^-avenasterol (*p* < 0.05).

#### 3.2.3. *β*-Carotene

*β*-Carotene is the most abundant provitamin A among carotenoids. Many fats and oils contain *β*-carotene, which contributes to the deep, intense orange red-color of various oils [6]. The *β*-carotene content ranged from 13.16 to 24.15 mg/kg (Table 4). Significantly higher contents (24.15 and 23.55 mg/kg for 2021 and 2022, respectively) were detected in oil samples extracted with 2-MeTHF compared to other samples (*p* < 0.05). Similarly, 2-MeTHF has also been reported to achieve a higher recovery of carotenoids compared to *n*-hexane [11,16,20,47]. For the harvest year, oils from 2022 had significantly higher *β*-carotene contents than those from 2021 across the solvents as shown in Table 4 (*p* < 0.05). Additionally, an examination of the relationship between *β*-carotene content and the color (especially the R-value) revealed a strong linear correlation with a high determination coefficient (R^2^ = 0.9725). Further analysis of variance showed that both solvent type and harvest year significantly affected the *β*-carotene content (*p* < 0.05).

### 3.3. Hierarchical Cluster Analysis

**Figure 4:**
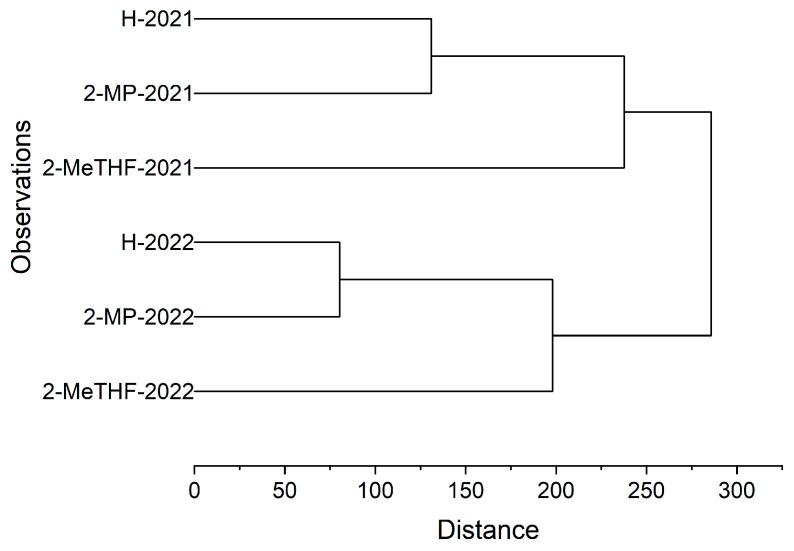
Dendrogram from hierarchical cluster analysis.

As illustrated in Figure 4, cluster analysis separated the oil samples into two distinct clusters corresponding to harvest years (2021 and 2022). This year-based separation highlights significant interannual variations in the physicochemical or compositional profiles of the oils, potentially attributable to environmental or agronomic conditions. Sub-clustering by solvent type further revealed that Hexane and 2-MP oils consistently co-clustered across harvest years, indicating shared characteristics. In contrast, 2-MeTHF-extracted oils formed an isolated cluster, reflecting unique compositional or functional divergence from other solvents. This divergence corroborates prior variance analyses of physicochemical properties and bioactive compounds, suggesting 2-MeTHF’s distinct extraction efficiency or chemical selectivity.

As for 2-MP, it rendered the oils’ comparable levels of physicochemical properties and active compounds to those of *n*-hexane-extracted oils. The observed clustering patterns, validated by distance metrics, confirm that harvest year and solvent type critically determine the oils’ physicochemical and bioactive compound profiles.

## 4. Conclusions

This study systematically analyzes the physicochemical properties of *A. lobatum* kernel oil extracted with bio-based and petroleum-derived solvents, demonstrating the viability of 2-MeTHF as a sustainable alternative to *n*-hexane and 2-MP. 2-MeTHF achieved a higher oil yield, surpassing *n*-hexane and 2-MP, while maintaining equivalent or improved quality metrics, including total tocols and *β*-carotene. The extracted oils exhibited favorable fatty acid profiles, particularly rich in oleic and linoleic acids, along with significant levels of tocopherols, tocotrienols, phytosterols, and *β*-carotene, which contribute to their nutritional and functional value. The study also revealed that both solvent type and harvest year significantly influenced the oxidative stability, color, and thermal properties of the oils. Meanwhile, 2-MP may be a comparative alternative to *n*-hexane. Overall, 2-MeTHF not only offers a more environmentally friendly extraction method but also maintains or enhances the quality of *A. lobatum* kernel oil, positioning it as a viable solvent for commercial edible oil production. Future research should focus on optimizing extraction conditions and exploring the potential of other green solvents or mixed green solvent systems to further enhance the sustainability and quality of oil extraction processes. Strategies such as refining or addition of antioxidants to address the susceptibility of 2-MeTHF-extracted oils to oxidize are another demanding challenge.

## Figures and Tables

**Table 1:** Physicochemical characteristics of *A. lobatum* kernel oil.

Variable	Oil Samples					
	Hexane-2021	2-MeTHF-2021	2-MP-2021	Hexane-2022	2-MeTHF-2022	2-MP-2022
Oil Yield (%)	24.71 ± 0.14 ^c^	27.60 ± 0.76 ^b^	22.74 ± 0.63 ^d^	28.12 ± 0.10 ^b^	29.77 ± 0.48 ^a^	25.71 ± 0.09 ^c^
Color (R and Y) Lovibond units 1” cell	R, 1.65; Y, 15	R, 18.05; Y, 39	R, 2.15; Y, 15	R, 1.20; Y, 18	R, 16.40; Y, 38	R, 1.40; Y, 19
5R + Y	23.25 ± 0.35 ^e^	129.25 ± 1.77 ^a^	25.75 ± 0.35 ^c^	24.00 ± 0.00 ^d^	120.00 ± 0.71 ^b^	26.00 ± 0.00 ^c^
*K*_232_	2.39 ± 0.25 ^c^	11.58 ± 0.54 ^a^	2.47 ± 0.02 ^c^	1.90 ± 0.07 ^c^	10.14 ± 0.75 ^b^	2.04 ± 0.13 ^c^
*K*_268_	0.18 ± 0.01 ^c^	2.65 ± 0.21 ^a^	0.22 ± 0.04 ^c^	0.12 ± 0.03 ^c^	2.46 ± 0.13 ^b^	0.14 ± 0.02 ^c^
AV (mg KOH/g)	1.21 ± 0.13 ^c^	3.75 ± 0.77 ^a^	0.75 ± 0.07 ^e^	0.81 ± 0.04 ^de^	2.57 ± 0.30 ^b^	0.85 ± 0.17 ^d^
*p*-AnV	2.61 ± 0.34 ^f^	183.97 ± 1.45 ^b^	4.48 ± 0.51 ^e^	11.76 ± 3.20 ^c^	201.00 ± 4.30 ^a^	8.59 ± 1.20 ^d^
OSI (h)	11.12 ± 0.39 ^d^	17.13 ± 1.32 ^c^	41.50 ± 2.58 ^a^	28.47 ± 3.87 ^b^	1.35 ± 0.51 ^e^	25.91 ± 2.10 ^b^
Lauric acid (C12:0)	0.61 ± 0.00 ^b^	0.61 ± 0.00 ^b^	0.61 ± 0.00 ^b^	0.68 ± 0.01 ^a^	0.67 ± 0.00 ^a^	0.68 ± 0.01 ^a^
Myristic acid (C14:0)	4.13 ± 0.04 ^b^	4.10 ± 0.04 ^b^	4.12 ± 0.05 ^b^	4.35 ± 0.02 ^a^	4.33 ± 0.03 ^a^	4.34 ± 0.03 ^a^
Palmitic acid (C16:0)	7.15 ± 0.07 ^a^	7.26 ± 0.08 ^a^	7.14 ± 0.08 ^a^	6.54 ± 0.05 ^b^	6.60 ± 0.08 ^b^	6.54 ± 0.03 ^b^
Palmitoleic acid (C16:1)	0.14 ± 0.01 ^a^	0.14 ± 0.01 ^a^	0.14 ± 0.01 ^a^	0.09 ± 0.00 ^b^	0.09 ± 0.00 ^b^	0.09 ± 0.01 ^b^
Margaric acid (C17:0)	0.04 ± 0.00 ^a^	0.04 ± 0.00 ^a^	0.04 ± 0.01 ^a^	0.05 ± 0.00 ^a^	0.05 ± 0.01 ^a^	0.05 ± 0.00 ^a^
Stearic acid (C18:0)	2.68 ± 0.01 ^b^	2.71 ± 0.01 ^b^	2.68 ± 0.00 ^b^	2.98 ± 0.01 ^a^	3.01 ± 0.02 ^a^	2.97 ± 0.00 ^a^
Oleic acid (C18:1n9c)	46.86 ± 0.20 ^a^	46.78 ± 0.31 ^a^	46.91 ± 0.13 ^a^	36.68 ± 0.02 ^b^	36.56 ± 0.09 ^b^	36.75 ± 0.05 ^b^
Linoleic acid (C18:2n6c)	36.8 ± 0.04 ^b^	36.76 ± 0.15 ^b^	36.75 ± 0.02 ^b^	46.51 ± 0.13 ^a^	46.55 ± 0.04 ^a^	46.47 ± 0.10 ^a^
Arachidic acid (C20:0)	0.27 ± 0.04 ^a^	0.27 ± 0.04 ^a^	0.27 ± 0.04 ^a^	0.28 ± 0.04 ^a^	0.28 ± 0.04 ^a^	0.28 ± 0.04 ^a^
Eicosenoic acid (C20:1)	0.24 ± 0.00 ^a^	0.24 ± 0.01 ^a^	0.24 ± 0.00 ^a^	0.23 ± 0.00 ^a^	0.23 ± 0.01 ^a^	0.22 ± 0.00 ^a^
Linolenic acid (C18:3n3)	0.62 ± 0.01 ^b^	0.63 ± 0.00 ^b^	0.63 ± 0.00 ^b^	1.10 ± 0.01 ^a^	1.11 ± 0.02 ^a^	1.10 ± 0.02 ^a^
Behenic acid (C22:0)	0.33 ± 0.01 ^b^	0.34 ± 0.00 ^b^	0.34 ± 0.00 ^b^	0.44 ± 0.01 ^b^	0.43 ± 0.00 ^b^	0.43 ± 0.00 ^b^
Lignoceric acid (C24:0)	0.12 ± 0.00 ^a^	0.12 ± 0.00 ^a^	0.12 ± 0.00 ^a^	0.09 ± 0.00 ^b^	0.09 ± 0.00 ^b^	0.09 ± 0.00 ^b^
SFAs	15.30 ± 0.18 ^a^	15.17 ± 0.49 ^a^	15.05 ± 0.49 ^a^	15.08 ± 0.50 ^a^	15.16 ± 0.52 ^a^	15.06 ± 0.45 ^a^
MUFAs	47.24 ± 0.20 ^a^	47.05 ± 0.14 ^a^	47.18 ± 0.04 ^a^	36.88 ± 0.17 ^b^	36.76 ± 0.07 ^b^	36.95 ± 0.20 ^b^
PUFAs	37.42 ± 0.03 ^a^	37.39 ± 0.15 ^a^	37.38 ± 0.03 ^a^	47.61 ± 0.14 ^b^	47.66 ± 0.06 ^b^	47.57 ± 0.12 ^b^
UFAs/SFAs	5.54 ± 0.08 ^a^	5.57 ± 0.18 ^a^	5.62 ± 0.18 ^a^	5.61 ± 0.18 ^a^	5.57 ± 0.19 ^a^	5.62 ± 0.16 ^a^
n-3/n-6	0.02 ± 0.00 ^a^	0.02 ± 0.00 ^a^	0.02 ± 0.00 ^a^	0.02 ± 0.00 ^a^	0.02 ± 0.00 ^a^	0.02 ± 0.00 ^a^
AI	0.29 ± 0.00 ^a^	0.29 ± 0.00 ^a^	0.29 ± 0.00 ^a^	0.29 ± 0.00 ^a^	0.29 ± 0.00 ^a^	0.29 ± 0.00 ^a^
TI	0.27 ± 0.01 ^a^	0.27 ± 0.00 ^a^	0.27 ± 0.00 ^a^	0.26 ± 0.00 ^a^	0.26 ± 0.01 ^a^	0.26 ± 0.00 ^a^
HH	7.03 ± 0.09 ^a^	6.98 ± 0.08 ^a^	7.05 ± 0.09 ^a^	7.20 ± 0.05 ^a^	7.16 ± 0.08 ^a^	7.20 ± 0.03 ^a^

Note: SFAs: saturated fatty acids, MUFAs: mono-unsaturated fatty acids, PUFAs: polyunsaturated fatty acids, UFAs: unsaturated fatty acids, AI: atherogenicity index, TI: thrombogenicity index, HH: hypo- and hyper-cholestrolemic index. Values are given as mean ± SD (*n* = 2 or 3). Different letters in the same row indicate significant differences (*p* ≤ 0.05).

**Table 2:** DSC parameters of crystallization curves for *A. lobatum* kernel oil at scanning rate 2 °C/min.

Parameters	Peaks	Hexane-2021	2-MeTHF-2021	2-MP-2021	Hexane-2022	2-MeTHF-2022	2-MP-2022
Onset temperature (°C)	Peak 1	−18.03 ± 2.69 ^a^	−19.64 ± 5.44 ^b^	−17.89 ± 0.35 ^a^	−18.48 ± 0.85 ^a^	−19.62 ± 2.40 ^b^	−18.53 ± 4.10 ^a^
Peak temperature (°C)		−18.36 ± 2.40 ^a^	−19.98 ± 5.16 ^cd^	−18.33 ± 0.21 ^a^	−18.99 ± 0.85 ^ab^	−20.19 ± 2.62 ^d^	−19.39 ± 1.48 ^bc^
Offset temperature (°C)		−19.19 ± 1.98 ^ab^	−20.63 ± 5.94 ^de^	−19.12 ± 0.07 ^a^	−19.82 ± 0.35 ^bc^	−21.03 ± 2.19 ^e^	−20.21 ± 0.85 ^cd^
Peak height (W/g)		0.13 ± 0.00 ^a^	0.07 ± 0.00 ^c^	0.08 ± 0.01 ^bc^	0.11 ± 0.01 ^ab^	0.07 ± 0.00 ^c^	0.07 ± 0.03 ^c^
Enthalpy (ΔH, J/g)		0.26 ± 0.35 ^a^	0.12 ± 0.60 ^a^	0.17 ± 0.10 ^a^	0.27 ± 0.44 ^a^	0.12 ± 0.17 ^a^	0.17 ± 0.12 ^a^
Onset temperature (°C)	Peak 2	−47.11 ± 0.13 ^a^	−50.22 ± 0.46 ^b^	−47.43 ± 0.23 ^a^	−51.74 ± 0.08 ^c^	−55.03 ± 0.32 ^d^	−51.81 ± 0.48 ^c^
Peak temperature (°C)		−48.63 ± 0.13 ^a^	−52.13 ± 0.16 ^b^	−49.02 ± 0.11 ^a^	−53.90 ± 0.37 ^c^	−57.89 ± 0.52 ^d^	−54.39 ± 0.20 ^c^
Offset temperature (°C)		−51.41 ± 0.30 ^a^	−54.96 ± 0.20 ^b^	−51.67 ± 0.24 ^a^	−57.63 ± 0.14 ^c^	−62.39 ± 0.31 ^c^	−57.63 ± 0.87 ^b^
Peak height (W/g)		0.54 ± 0.03 ^a^	0.38 ± 0.05 ^ab^	0.43 ± 0.04 ^ab^	0.40 ± 0.07 ^ab^	0.22 ± 0.02 ^c^	0.32 ± 0.10 ^bc^
Enthalpy (ΔH, J/g)		41.57 ± 1.24 ^a^	33.58 ± 0.69 ^b^	33.43 ± 1.90 ^b^	36.82 ± 3.28 ^ab^	26.71 ± 1.48 ^c^	33.30 ± 4.38 ^b^

Note: Different letters in the same row indicate significant differences (*p* ≤ 0.05).

**Table 3:** DSC parameters of melting curves for *A. lobatum* kernel oil at scanning rate 2 °C/min.

Parameters	Onset Temperature (°C)	Peak Temperature (°C)	Offset Temperature (°C)	Peak Height (W/g)	Enthalpy (ΔH, J/g)
Hexane-2021	−25.49 ± 0.04 ^a^	−21.76 ± 0.18 ^a^	−20.54 ± 0.28 ^a^	−0.23 ± 0.02 ^c^	14.78 ± 0.30 ^abc^
2-MeTHF-2021	−28.85 ± 1.29 ^b^	−24.12 ± 0.71 ^b^	−22.41 ± 0.69 ^bc^	−0.12 ± 0.03 ^a^	10.42 ± 1.45 ^c^
2-MP-2021	−25.84 ± 0.56 ^a^	−22.22 ± 0.40 ^a^	−20.92 ± 1.17 ^ab^	−0.18 ± 0.00 ^b^	12.66 ± 4.65 ^bc^
Hexane-2022	−30.62 ± 0.93 ^b^	−25.42 ± 0.32 ^b^	−23.53 ± 0.29 ^cd^	−0.19 ± 0.02 ^bc^	20.94 ± 0.19 ^a^
2-MeTHF-2022	−34.03 ± 0.46 ^c^	−27.58 ± 0.15 ^c^	−24.87 ± 0.30 ^d^	−0.08 ± 0.00 ^a^	9.29 ± 0.27 ^c^
2-MP-2022	−30.47 ± 2.31 ^c^	−25.40 ± 1.05 ^b^	−23.35 ± 0.81 ^cd^	−0.18 ± 0.02 ^b^	19.35 ± 4.42 ^ab^

Note: Different letters in the same column indicate significant differences at (*p* ≤ 0.05).

**Table 4:** The contents of tocopherols, tocotrienols, phytosterols, and *β*-carotene in *A. lobatum* kernel oil (mg/kg).

Variable	Oil Samples					
	Hexane-2021	2-MeTHF-2021	2-MP-2021	Hexane-2022	2-MeTHF-2022	2-MP-2022
Tocopherols						
*α*	172.48 ± 0.90 ^a^	157.35 ± 0.31 ^b^	172.38 ± 7.97 ^a^	138.93 ± 6.90 ^c^	136.11 ± 1.90 ^cd^	126.61 ± 0.75 ^d^
*β*	353.13 ± 2.79 ^b^	321.60 ± 0.09 ^c^	358.87 ± 17.39 ^b^	390.85 ± 18.04 ^a^	386.07 ± 0.25 ^a^	356.77 ± 0.48 ^b^
*γ*	42.21 ± 1.58 ^a^	38.07 ± 1.27 ^ab^	39.12 ± 1.04 ^ab^	42.04 ± 3.78 ^a^	42.71 ± 2.06 ^a^	35.35 ± 0.59 ^b^
*δ*	396.32 ± 1.77 ^c^	359.68 ± 0.27 ^d^	396.58 ± 19.74 ^c^	512.76 ± 25.80 ^a^	505.79 ± 0.02 ^a^	463.85 ± 0.19 ^b^
Tocotrienols						
*α*	6.53 ± 1.39 ^a^	10.58 ± 5.13 ^a^	8.10 ± 1.15 ^a^	13.64 ± 5.27 ^a^	25.76 ± 18.39 ^a^	15.33 ± 1.85 ^a^
*γ*	4.33 ± 0.19 ^ab^	5.56 ± 2.66 ^ab^	4.45 ± 0.69 ^ab^	7.05 ± 2.72 ^ab^	14.63 ± 9.62 ^a^	0.31 ± 0.04 ^b^
*δ*	1.49 ± 0.08 ^b^	2.32 ± 0.83 ^b^	2.02 ± 0.33 ^b^	2.68 ± 0.98 ^b^	7.77 ± 4.72 ^a^	0.26 ± 0.16 ^b^
Total tocols	976.49 ± 8.55 ^bc^	895.15 ± 9.95 ^c^	981.52 ± 43.98 ^bc^	1107.95 ± 63.50 ^a^	1118.83 ± 36.41 ^a^	998.48 ± 0.82 ^b^
Phytosterols						
Brassicasterol	65.56 ± 0.80 ^c^	66.65 ± 0.49 ^bc^	69.73 ± 0.38 ^a^	65.48 ± 0.67 ^c^	68.39 ± 0.87 ^ab^	67.11 ± 1.25 ^bc^
Campesterol	326.11 ± 8.64 ^a^	295.00 ± 7.07 ^a^	320.89 ± 8.34 ^a^	348.97 ± 12.69 ^a^	338.20 ± 2.55 ^a^	314.23 ± 69.62 ^a^
Campestanol	23.00 ± 0.00 ^a^	24.74 ± 1.78 ^a^	22.87 ± 0.19 ^ab^	21.56 ± 2.03 ^ab^	22.17 ± 2.59 ^ab^	18.42 ± 2.01 ^b^
Stigmasterol	21.06 ± 1.33 ^b^	24.39 ± 0.86 ^a^	20.10 ± 0.15 ^b^	13.66 ± 0.48 ^cd^	15.09 ± 1.29 ^c^	12.56 ± 0.79 ^d^
Clerosterol	19.38 ± 1.95 ^c^	26.18 ± 1.66 ^b^	18.00 ± 0.00 ^c^	24.89 ± 0.16 ^b^	32.09 ± 2.69 ^a^	18.86 ± 0.93 ^c^
*β*-Sitosterol	1358.34 ± 2.34 ^a^	1269.84 ± 21.44 ^c^	1323.08 ± 11.42 ^ab^	1285.37 ± 10.43 ^bc^	1205.01 ± 7.09 ^d^	1320.44 ± 27.67 ^ab^
Δ^5^-Avenasterol	112.24 ± 1.07 ^a^	76.60 ± 2.26 ^c^	105.62 ± 3.36 ^b^	57.49 ± 0.73 ^d^	48.57 ± 3.63 ^e^	55.03 ± 1.36 ^d^
Stigmastadienol	116.40 ± 6.50 ^a^	103.88 ± 0.17 ^bc^	114.91 ± 5.78 ^ab^	90.50 ± 4.94 ^de^	80.82 ± 1.15 ^e^	94.43 ± 5.05 ^cd^
Stigmastenol	208.40 ± 4.80 ^a^	167.80 ± 3.11 ^b^	201.35 ± 1.91 ^a^	146.25 ± 6.71 ^c^	139.62 ± 0.54 ^c^	141.67 ± 2.36 ^c^
Δ^7^-Avenasterol	16.83 ± 1.66 ^bc^	21.74 ± 2.47 ^a^	17.81 ± 1.15 ^bc^	15.09 ± 0.12 ^bc^	14.86 ± 1.21 ^c^	18.62 ± 0.54 ^ab^
Total sterols	2267.33 ± 3.30 ^a^	2076.83 ± 14.38 ^c^	2214.37 ± 13.25 ^b^	2069.26 ± 8.85 ^c^	1964.80 ± 2.54 ^d^	2061.36 ± 39.83 ^c^
*β*-carotene	13.16 ± 0.02 ^d^	24.15 ± 0.44 ^a^	13.45 ± 0.18 ^c^	14.55 ± 0.01 ^c^	23.55 ± 0.03 ^a^	15.02 ± 0.04 ^b^

Note: different letters in the same row indicate significant differences (*p* ≤ 0.05).

## Data Availability

The original contributions presented in the study are included in the article/Supplementary Materials, further inquiries can be directed to the corresponding author.

## Supplementary Materials

The following supporting information can be downloaded at https://www.mdpi.com/article/10.3390/foods14101682/s1. Table S1. Relevant physicochemical properties of solvents; Figure S1. Heatmap with dendrogram of the oils using the collected fatty acid data. Figure S2. FT-IR spectra of *A. lobatum* kernel oil.
