# Supplementary material for: Sustainable Extraction of Actinostemma lobatum Kernel Oil by 2-Methyltetrahydrofuran: A Comparative Study on Physicochemical Properties and Bioactive Compounds Against Petro-Sourced Solvents
Source: Foods. 2025 May 9;14(10):1682. doi: 10.3390/foods14101682 (PMC12111321; doi:10.3390/foods14101682)
Supplement: Supplementary file 1 [file foods-14-01682-s001.zip › Highlights.pdf]

Highlights:

- 2-MeTHF yields significantly higher oil content than *n*-hexane and 2-MP.
- Oils extracted with 2-MeTHF show comparable or superior bioactive compound content.
- Low DSC peak temperature, T_{on}, and T_{off} were observed for 2-MeTHF-extracted oils.
- Both the solvent type and harvest year affected the oil quality.
- 2-MeTHF seems to be a sustainable, efficient alternative for oil extraction.
