# Supplementary material for: Sustainable Extraction of Actinostemma lobatum Kernel Oil by 2-Methyltetrahydrofuran: A Comparative Study on Physicochemical Properties and Bioactive Compounds Against Petro-Sourced Solvents
Source: Foods. 2025 May 9;14(10):1682. doi: 10.3390/foods14101682 (PMC12111321; doi:10.3390/foods14101682)
Supplement: Supplementary file 1 [file foods-14-01682-s001.zip › Supplementary materails.pdf]

Sustainable Extraction of *Actinostemma lobatum* Kernel Oil by 2-Methyltetrahydrofuran: A Comparative Study on Physicochemical Properties and Bioactive Compounds Against Petro-sourced Solvents

Liyou Zheng [1,†], Hongyan Guo [1,†], Haozhi Song [1], Miao Yu [2], Mengxi Xie [2], Sameh A. Korma [3, 4*], and Tao Zhang [5,*]

[1] School of Biological and Food Engineering, Anhui Polytechnic University, Wuhu 241000, China; zhengliyou@ahpu.edu.cn (L.Z.); 2106032@mail.ahpu.edu.cn (H.G.)

[2] Institute of Food and Processing, Liaoning Academy of Agricultural Sciences, Shenyang 110161, China; jannytiti@163.com (M.Y.); moor1112@163.com (M.X.)

[3] Department of Food Science, Faculty of Agriculture, Zagazig University, Zagazig 44519, Egypt

[4] School of Food Science and Engineering, South China University of Technology, Guangzhou 510641, China

[5] College of Food Science and Technology, Huazhong Agricultural University, Wuhan 430070, China

† These authors contributed equally to this work.

* Correspondence: t.zhang@mail.hzau.edu.cn (T.Z.); sameh.hosny@zu.edu.eg (S.A.K.); Tel.: +86-15190271919 (T.Z.); +86-15818114375 (S.A.K.)

Table S1. Relevant physicochemical properties of solvents[1-5].

Properties	*n*-Hexane	2-MeTHF	2-MP
Molecular formula	C_6H_{14}	$C_5H_{10}O$	C_6H_{14}
Molecular weight (g/mol)	86.18	86.13	86.18
Density (g/mL)	0.65	0.85	0.65
Boiling point (◦C)	68.7	80.2	60.2
Partition coefficient (Log p) [a]	4	1.85	3.2
Resource	Petro-sourced	Bio-based	Petro-sourced

Note: a, a measure of lipophilicity obtained from PubChem

(https://pubchem.ncbi.nlm.nih.gov/)

Section S1

The broad peak centered at 3458 cm^{-1} might be linked to the stretching of O-H bonds present in fatty acids , the presence of hydroxyl groups (-OH) due to hydroperoxide in the oil [6], or the stretching of the C=O bond of triacylglycerols [7]. The peak at 3007 cm^{-1} was associated with the C–H stretching vibration of the carbon-carbon cis double bond, specifically representing unsaturated fatty acids [8]. The peaks observed at 2926 and 2854 cm^{-1} correspond to the asymmetric and symmetric stretching of the C-H bond of aliphatic CH_2 functional groups present in fatty acids or triacylglycerols, respectively [9]. As shown in the spectra, no peaks at 2926 cm^{-1} were detected in the oils extracted from seeds cultivated in 2021 or in the oil sample (2-MP-2022). Strong carbonyl stretching vibrations of the –C=O (ester group) of aliphatic esters resulted in a distinct peak at 1745 cm^{-1}[8]. The shoulder at 1645 cm^{-1} was associated with *cis* carbon-carbon double band (C=C) stretching vibrations of the *cis*-olefins [7]. The vibration peak at 1460 was associated with the scissor and/or deformation of methyl (–CH_3) and methylene (–CH_2) groups present in the oil. The peak at 1373 cm^{-1} was attributed to the symmetric bending of –C-H (CH_3). The shoulder peaks at 1236 and 1099 cm^{-1}, along with a strong peak at 1165 cm^{-1}, were mainly due to the stretching vibrations of the –C–O (ester group) [7]. However, Han et al. (2020) indicated that the peaks at 1240 and 1165 cm^{-1} were due to the bending of –C–H (CH_2), while the peak at 1099 cm^{-1} may assigned to the stretching of the –C–O bonds of aliphatic esters . The peak at 723 cm^{-1} was attributed to the combination or overlapping of –CH_2 rocking and out-of-plane vibration of *cis*-disubstituted olefins *cis*-HC=CH [7,8].

References

[1] Trad, S., Chaabani, E., Aidi Wannes, W., Dakhlaoui, S., Nait Mohamed, S., Khammessi, S., et al. (2023). Quality of edible sesame oil as obtained by green solvents: In silico versus experimental screening approaches. *Foods, 12*(17), 3263. https://doi.org/10.3390/foods12173263.

[2] Rapinel, V., Claux, O., Abert-Vian, M., McAlinden, C., Bartier, M., Patouillard, N., et al. (2020). 2-Methyloxolane (2-MeOx) as sustainable lipophilic solvent to substitute hexane for green extraction of natural products. Properties, applications, and perspectives. *Molecules, 25*(15), 3417. https://doi.org/10.3390/molecules25153417.

[3] Claux, O., Rapinel, V., Goupy, P., Patouillard, N., Vian, M. A., Jacques, L., et al. (2021). Dry and aqueous 2-methyloxolane as green solvents for simultaneous production of soybean oil and defatted meal. *ACS Sustainable Chemistry and Engineering, 9*(21), 7211-7223. https://doi.org/10.1021/acssuschemeng.0c09252.

[4] Cravotto, C., Claux, O., Bartier, M., Fabiano-Tixier, A.-S., & Tabasso, S. (2023). Leading edge technologies and perspectives in industrial oilseed extraction. *Molecules, 28*(16), 5973. https://doi.org/10.3390/molecules28165973.

[5] Sicaire, A.-G., Vian, M., Fine, F., Joffre, F., Carré, P., Tostain, S., et al. (2015). Alternative bio-based solvents for extraction of fat and oils: Solubility prediction, global yield, extraction kinetics, chemical composition and cost of manufacturing. *International Journal of Molecular Sciences, 16*(4), 8430-8453. https://doi.org/10.3390/ijms16048430.

[6] Rajagukguk, Y. V., Islam, M., Grygier, A., & Tomaszewska-Gras, J. (2023). Thermal and spectroscopic profiles variation of cold-pressed raspberry seed oil studied by DSC, UV/VIS, and FTIR techniques. *Journal of Food Composition and Analysis, 124*, 105723. https://doi.org/10.1016/j.jfca.2023.105723.

[7] Mohammadi, N., Ostovar, N., & Granato, D. (2023). Pyrus glabra seed oil as a new source of mono and polyunsaturated fatty acids: Composition, thermal, and FTIR spectroscopic characterization. *LWT-Food Science and Technology, 181*, 114790. https://doi.org/10.1016/j.lwt.2023.114790.

[8] Rodríguez, M. E., Rikal, L., Schneider-Teixeira, A., Deladino, L., & Ixtaina, V. (2023). Extraction method impact on the physicochemical characteristics of lipids from chia nutlets applicable to long-term storage studies. *Food Chemistry, 427*, 136706. https://doi.org/10.1016/j.foodchem.2023.136706.

[9] Embaby, H. E., Miyakawa, T., Hachimura, S., Muramatsu, T., Nara, M., & Tanokura, M. (2022). Crystallization and melting properties studied by DSC and FTIR spectroscopy of goldenberry (*Physalis peruviana*) oil. *Food Chemistry, 366*, 130645. https://doi.org/10.1016/j.foodchem.2021.130645.

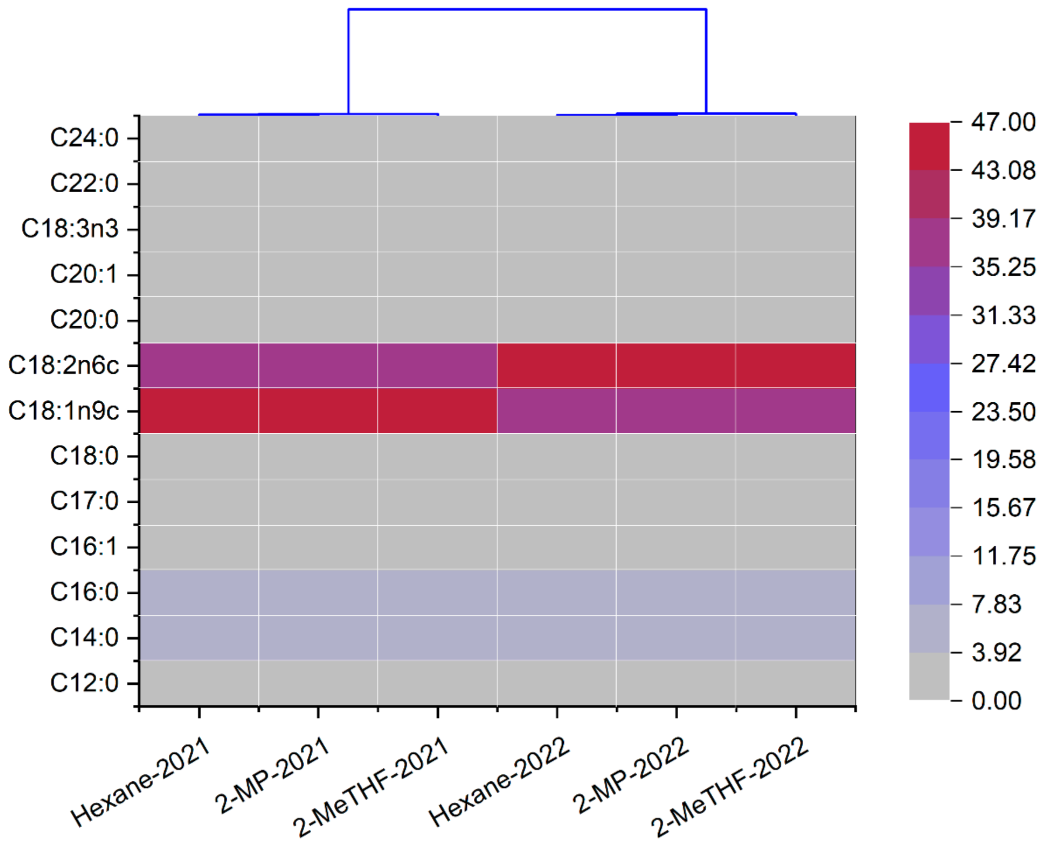

Figure S1. Heatmap with dendrogram of the oils using the collected fatty acid data.

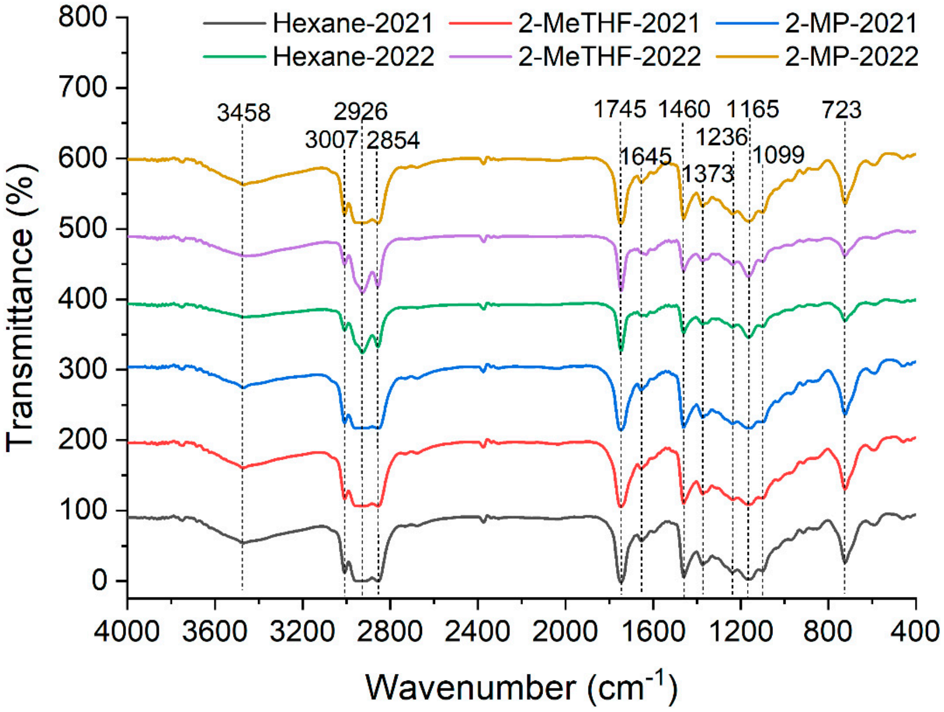

Figure S2. FT-IR spectra of *A. lobatum* kernel oil.
