# Supplementary figures and images for: Sustainable Extraction of Actinostemma lobatum Kernel Oil by 2-Methyltetrahydrofuran: A Comparative Study on Physicochemical Properties and Bioactive Compounds Against Petro-Sourced Solvents
Source: Foods. 2025 May 9;14(10):1682. doi: 10.3390/foods14101682 (PMC12111321; doi:10.3390/foods14101682)

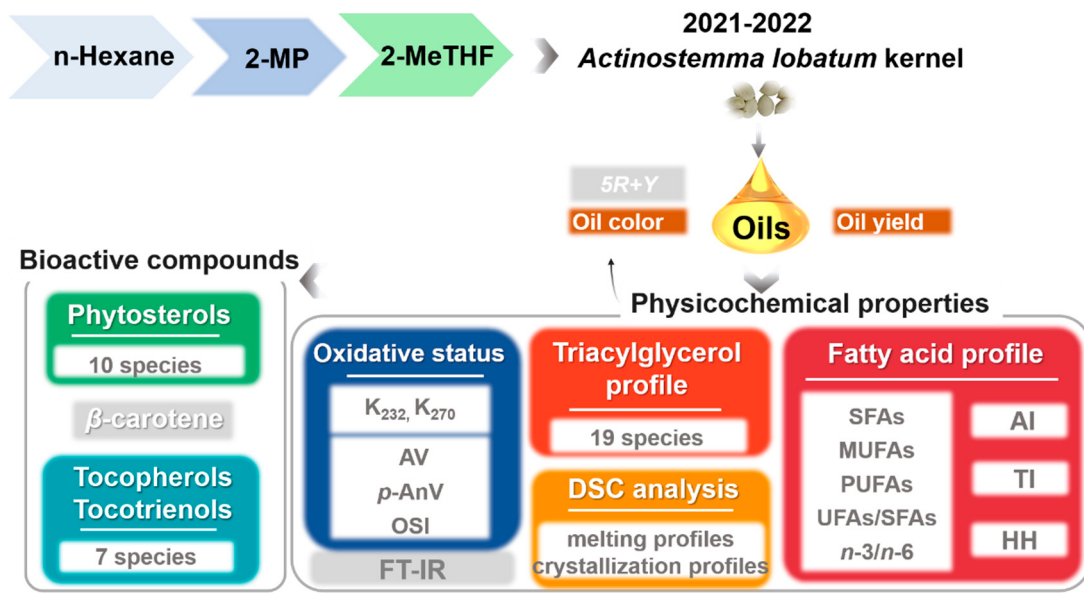

Supplement: Supplementary file 1 [file foods-14-01682-s001.zip › Graphical abstract.pdf]
